# Human sensitivity to vertical self-motion

**DOI:** 10.1007/s00221-013-3741-8

**Published:** 2013-10-25

**Authors:** Alessandro Nesti, Michael Barnett-Cowan, Paul R. MacNeilage, Heinrich H. Bülthoff

**Affiliations:** 1 Department of Human Perception, Cognition and Action, Max Planck Institute for Biological Cybernetics, Spemannstraße 38, 72076 Tübingen, Germany; 2 Department of Kinesiology, University of Waterloo, Waterloo, ON N2L 3G1 Canada; 3 German Center for Vertigo and Balance Disorders, Ludwig Maximilians University Hospital of Munich, Marchioninistr. 23, 81377 Munich, Germany; 4 Department of Brain and Cognitive Engineering, Korea University, Seoul, 136-71 Korea

**Keywords:** Differential threshold, Psychophysics, Otolith, Self-motion perception, Vestibular, Heave, Gravity

## Abstract

Perceiving vertical self-motion is crucial for maintaining balance as well as for controlling an aircraft. Whereas heave absolute thresholds have been exhaustively studied, little work has been done in investigating how vertical sensitivity depends on motion intensity (i.e., differential thresholds). Here we measure human sensitivity for 1-Hz sinusoidal accelerations for 10 participants in darkness. Absolute and differential thresholds are measured for upward and downward translations independently at 5 different peak amplitudes ranging from 0 to 2 m/s^2^. Overall vertical differential thresholds are higher than horizontal differential thresholds found in the literature. Psychometric functions are fit in linear and logarithmic space, with goodness of fit being similar in both cases. Differential thresholds are higher for upward as compared to downward motion and increase with stimulus intensity following a trend best described by two power laws. The power laws’ exponents of 0.60 and 0.42 for upward and downward motion, respectively, deviate from Weber’s Law in that thresholds increase less than expected at high stimulus intensity. We speculate that increased sensitivity at high accelerations and greater sensitivity to downward than upward self-motion may reflect adaptations to avoid falling.

## Introduction

Humans can move in three dimensions. Compared to fore-aft and lateral movements (also called surge and sway, respectively), vertical self-motion (or heave) is particularly physically and ecologically constrained due to the constant force of gravity. The downward force of gravity is equivalent to an upward acceleration that is detected by the otolith organs of the vestibular system. At rest, the otoliths indicate an upward acceleration at the rate of 9.8 m/s^2^. But when at rest we do not feel as though we are moving, suggesting that the brain must compensate for gravity’s constant influence. Despite this compensation, the presence of a non-zero pedestal stimulus could lead to asymmetric sensitivity to earth vertical motion. In particular, it is not yet known whether sensitivities to upward and downward self-motion differ. Here we investigate this question by measuring how sensitivity depends on the magnitude and direction of vertical acceleration.

Human self-motion perception arises from central processing of sensory information from the visual, vestibular, auditory, and somatosensory systems. The first step to characterize this complex perceptual process is to understand how these various sources individually contribute to the subjective representation of physical motion. Such characterization finds immediate application in the field of vehicle simulation and in clinical assessment of balance disorders. Motion algorithms for dynamic simulators rely on human self-motion perception knowledge to provide, within their limited workspace, the most realistic motion sensation. Predicting the perception of vertical self-motion is especially important for flight simulation, particularly during takeoff and landing maneuvers. In the medical field, current protocols for diagnosing orientation perception disorders rely on measuring oculomotor reflexes (Bárány 1921; Halmagyi and Curthoys 1988). However, perception and reflexes do not always correlate (Kanayama et al. 1995; Merfeld et al. 2005a, b). Psychophysical measurements are therefore a helpful tool for localizing the disorder source (Merfeld et al. 2010; Agrawal et al. 2013).

A common method for investigating human self-motion perception is to measure perceptual thresholds by asking participants to make judgments based on the provided motion stimulation. These experimental studies can be divided into two main categories: estimation of absolute thresholds (the smallest detectable *level* of a stimulus intensity) and estimation of differential thresholds (the smallest detectable *change* in stimulus intensity). To measure absolute thresholds, participants usually perform either a detection task (report the presence of motion) or a direction discrimination task (report motion direction, sometimes also referred to as direction recognition task). To measure differential thresholds, participants perform an amplitude discrimination task (discriminate between two different movements).

Whereas detection and direction discrimination thresholds for human self-motion have been exhaustively studied, little work has been done in investigating our ability to discriminate vertical self-movements over different ranges of motion intensities. Human differential thresholds to linear self-motion have been investigated by Naseri and Grant (2012) over a range of 0.5–2 m/s^2^ for surge motion and by Zaichik et al. (1999) over a range of 0–0.6 m/s^2^ for surge, sway, and heave motion. Their works show that differential thresholds increase with stimulus intensity following Weber’s perceptual law (Fechner 1860). According to this fundamental law of psychophysics, the change in a stimulus that is just noticeable (differential threshold) is a constant ratio of the original stimulus. However, this was not confirmed by MacNeilage et al. (2010), who found no significant change in sensitivity for surge and heave motion over a range of 0–0.3 m/s^2^. Thus, it remains unresolved whether perceived linear self-motion (1) is independent from stimulus intensity; (2) follows Weber’s law; (3) follows a different (nonlinear) law. To assess these competing hypotheses, the present work sets out to describe differential thresholds for heave motion in the absence of visual cues as a function of motion intensity.

Assessing whether or not perceived self-motion follows a Weber’s law is important as many current models used to mathematically describe the process of self-motion perception (Borah et al. 1988; Bos and Bles 2002; Zupan et al. 2002) assume that sensitivity to supra-threshold self-motion is not affected by motion intensity once absolute threshold is overcome. This is true for the vestibular ocular reflex (VOR), which maintains a constant level of accuracy and precision over a wide range of motion intensities (Pulaski et al. 1981; Weber et al. 2008). The VOR, elicited by the vestibular system in response to head-in-space motion, is one of the vestibular mechanisms that maintain gaze and postural stability and, like self-motion perception, may be modulated from higher level neural processes. However, vestibular perception and action employ qualitatively different mechanisms (Barnett-Cowan et al. 2005; Merfeld et al. 2005a, b; Bertolini et al. 2011). If psychophysical evidence indicates a nonlinear relationship between self-motion perception and stimulus intensity, the accuracy of self-motion perception models over a wide motion range would benefit greatly from the implementation of differential thresholds.

With this work, we also address the question of asymmetries in motion perception between upward and downward movements. Evidence of asymmetry in vertical self-motion perceptual thresholds was previously reported by Benson et al. (1986) for participants lying on the back, who measured perceptual thresholds for vertical translations in head coordinates. They observed that movements in the footward direction are more readily perceived than movements in the headward direction. Notice that for participants lying on the back, the effect of gravity on head-relative heave motion is symmetric, suggesting that gravity is not responsible for vertical perceptual asymmetries. However, these results conflict with earlier (Melvill Jones and Young 1978) and more recent (MacNeilage et al. 2010; Roditi and Crane 2012) reports, where no significant differences are reported between opposite direction movements along the vertical axis. Determining whether discrepancies in vertical self-motion sensitivity exist is not only important to consider when designing motion control algorithms, such a discrepancy would yield insight into the dynamics of the inertial sensors and/or central processing of self-motion information. Asymmetries in the perception of upward and downward motion would reflect high-level processes in the central neural system (e.g., higher sensitivity to downward movements to prevent falls). Alternatively, similar sensitivities would suggest that human self-motion perception perfectly compensates for gravity both at rest and during motion. In this work, upward and downward sensitivity will be compared not only for movements close to absolute threshold but over a wide range of motion.

## Methods

### Participants

Ten subjects (3 females; aged 20–31 years), nine naïve and one author (MB-C), participated in the study and gave their informed written consent in accordance with the ethical standards specified by the 1964 Declaration of Helsinki prior to their inclusion in the study. Participants reported having no vestibular or other neurological disorders and no susceptibility to motion sickness.

### Setup

**Fig. 1:**
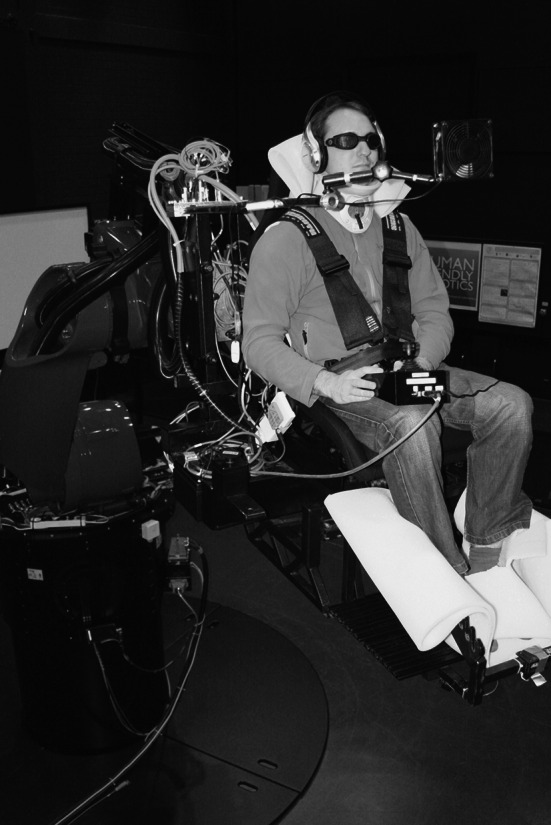
Experimental setup

The experiment was conducted using the Max Planck Institute CyberMotion Simulator. This motion simulator is based on a 6-degrees-of-freedom anthropomorphic robot arm and can provide a large variety of motion stimuli, with a maximal vertical displacement of about 1.4 m and a maximal linear acceleration of about 5 m/s^2^. Further details on its hardware and software specifications are available (Robocoaster, KUKA Roboter GmbH, Germany; Teufel et al. 2007; Barnett-Cowan et al. 2012). Participants were seated in a chair with a 5-point harness (Fig. 1). They wore light-proof goggles to eliminate visual information, as well as ear plugs (SNR = 33, NRR = 29) and headphones with acoustic white noise played back during the movements to eliminate external auditory cues from the simulator motors. None of the participants reported that the motor noise was heard during the experiment. To mask possible air movement cues during the motion, participants wore long trousers and sleeves, and a fan was installed in front of them. The seat and the feet of the participants were covered with foam to mask vibrations of the simulator. The use of a neck brace, combined with careful instruction to maintain an upright position, was assumed to minimize head movements. Head motion was therefore not recorded.

### Procedure

**Fig. 2:**
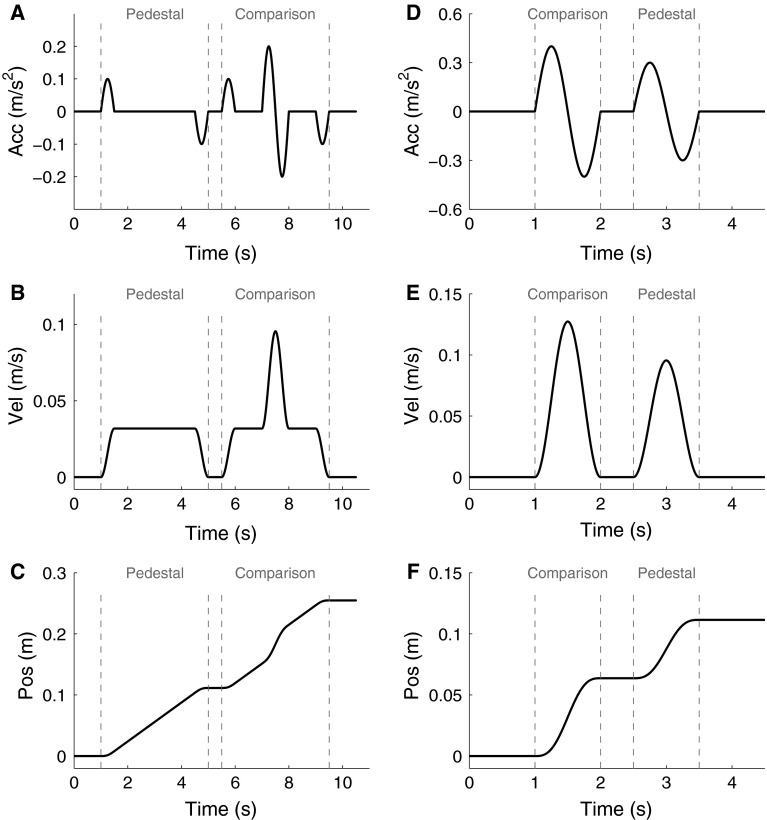
Acceleration, velocity, and position traces for a baseline condition trial (**a**, **b**, **c**, respectively) and for a trial with 0.3 m/s^2^ pedestal (**d**, **e**, **f**, respectively)

Each trial was composed of two consecutive vertical movements in the same direction. One of these movements remained unchanged in every trial (pedestal stimulus), while the second movement (comparison stimulus) systematically varied in amplitude. Accelerations during each interval were single-cycle sinusoidal profiles with 1 s duration (i.e. 1 Hz, see Fig. 2) with amplitudes ranging from 0 to 3.3 m/s^2^. This allows for comparison with previous research (Benson et al. 1986; MacNeilage et al. 2010; Naseri and Grant 2012; Roditi and Crane 2012) and is within the frequency range of flight simulations. Pedestal and comparison stimulus order was randomized to avoid order effects and complications due to motion after-effect (i.e., the influence of a previous motion on the next motion, Crane 2012).

Participants initiated each trial with a button press and, after a 1-s pause, the movement began. The two movements were separated by a 0.5-s interval where no motion was provided. After the second movement ended, participants were instructed to indicate via button press “which movement was stronger, the first or second, in terms of highest acceleration, velocity, and covered distance”. After answering in this two-interval forced-choice task (2IFC), they were moved back to the starting position with a slow, but supra-threshold movement involving all the simulator joints. No feedback was given.

The experiment consisted of five sessions lasting approximately 1.5 h each and was conducted on separate days. In each session, the absolute or differential threshold was assessed for two pedestal amplitudes with separate ~40-min blocks for upward and downward directions with breaks every 15 min to avoid fatigue. Peak pedestal amplitudes were 0 (baseline, see below), 0.3, 1.1, 1.6, or 2 m/s^2^. The 0.3 m/s^2^ pedestal was chosen so to allow comparison with MacNeilage et al. (2010), while the irregular spacing reflects our interest for highest motion intensities and was obtained with the following formula:

1 $$\varvec{p} = \frac{{\ln (1 + \varvec{x})}}{\ln (4)} \times (2 - 0.3) + 0.3$$

where ***p*** is the 4-element vector of pedestal intensities and ***x*** is a vector of 4 linearly spaced values between 0 and 3. Each pedestal was tested in the upward and downward direction separately, for a total of 10 conditions per participant.

The two baseline conditions (pedestal of 0 m/s^2^) employ slightly different methods to measure absolute rather than differential thresholds. In these conditions, movements were superimposed on a constant velocity translation in the same direction to avoid additional cues from motor activation (Fig. 2a). Velocity was initially increased following a half raised cosine profile for 0.5 s. Then, after 3 s of constant velocity motion, velocity was decreased back to zero with the other half of the original raised cosine profile. During the constant velocity phase, the 1 Hz sinusoidal acceleration profile was superimposed, with a time offset randomly selected from a list of 3 possible values: 0.5, 1 or 1.5 s. In this condition, participants’ were asked to report which of the two intervals had a superimposed acceleration, equivalent to a 2-interval detection task. Note, only 6 of the 10 participants completed this baseline condition. The other 4 participants were not available for further testing.

**Fig. 3:**
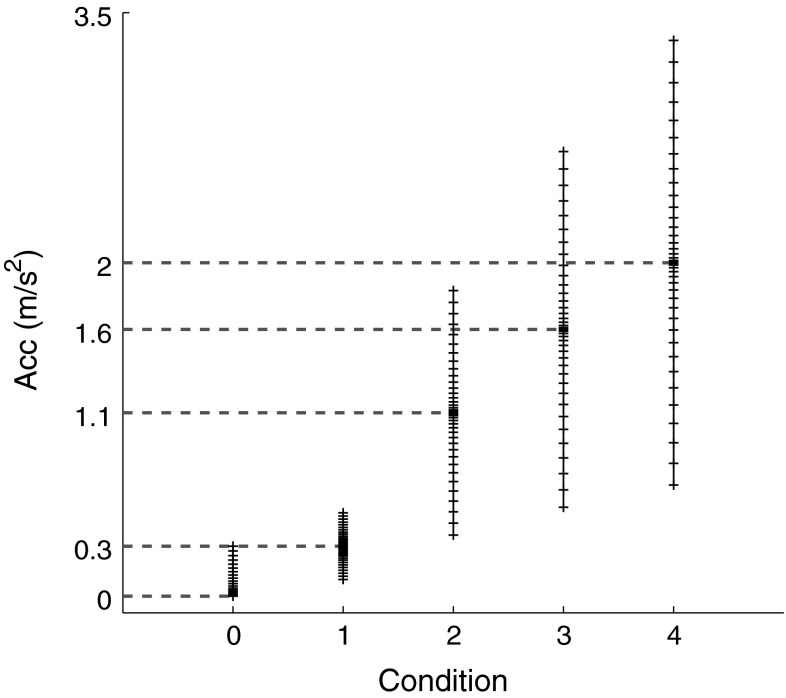
Graphical representation of the peak amplitude of the comparison stimuli used. The *gray dotted lines* indicate the pedestal intensities around which the set of stimuli was selected for each condition. *Each tick marks* a possible acceleration value to be presented

For each pedestal, 40 comparison stimuli were symmetrically distributed about each pedestal according to Eq. 2:

2 $$\varvec{c} = \left[ {1 \pm \frac{{\varvec{s}^{2} }}{{\frac{2}{3} \times p^{2} }}} \right] \times p$$

where ***c*** is the vector of comparison stimuli intensities, *p* is the pedestal stimulus intensity, and ***s*** is a 21-element vector linearly spaced between 0 and 2/3 × *p*. The resulting stimuli are in a range of ±67 % of the pedestal intensity, with higher stimulus density near the pedestal (Fig. 3). In the baseline condition, 21 stimuli were similarly placed between 0 and 0.3 m/s^2^.

**Fig. 4:**
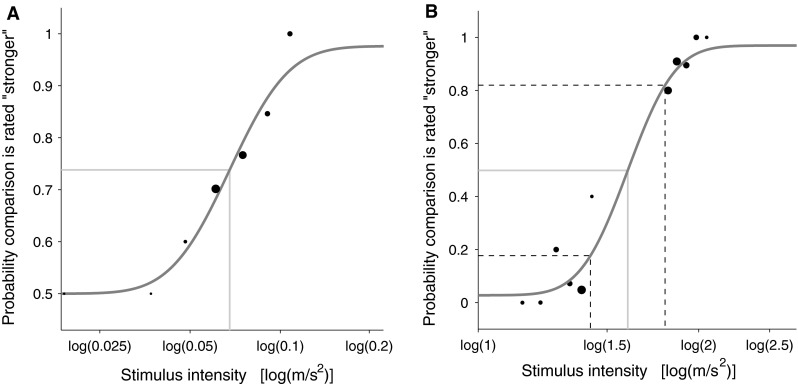
Psychometric functions fit (*gray line*) in log stimulus space to the data (*black dots*) obtained for one participant in the baseline condition (**a**) and for a pedestal of 1.6 m/s^2^ (**b**). The probability of rating the comparison stimulus as stronger than the pedestal is on the *y* axis. The *light gray line* and the *black dashed line* represent the mean and the standard deviation of the fitted cumulative Gaussian, respectively. **a** The *gray line* represents the stimulus that corresponds to the participant’s absolute threshold. **b** The *black dashed lines* represent stimuli that are one standard deviation weaker (*left dashed line*) or stronger (*right dashed line*) than the pedestal. According to our definition of differential threshold, the region on the *x* axis between the *black dotted lines* encloses stimuli that cannot be distinguished from the pedestal

The percentage of correct answers as a function of motion amplitude was fit with a continuous psychometric function (Kontsevich and Tyler 1999; Tanner 2008). The psychometric function was modeled as a cumulative normal distribution (Fig. 4). Two lapse parameters limited to 5 % were included into the fit to account for the possibility of accidentally pressing the wrong button even if the direction was correctly perceived. It has been shown that this can significantly improve the fit (Wichmann and Hill 2001). The fitting was performed in logarithmic stimulus space, a choice motivated by the proportional decrease in self-motion sensitivity with increasing stimulus intensity (Mallery et al. 2010; Naseri and Grant 2012; Zaichik et al. 1999) and because it has already been adopted in previous studies (Soyka et al. 2011, 2012).

Example psychometric fits are illustrated in Fig. 4. In all but the baseline conditions (Fig. 4b), the inflection point of the cumulative Gaussian corresponds to chance performance, i.e., 0.5 probability^1^ of answering correctly. The standard deviation corresponds to a change in the stimulus amplitude that increases the performance to 0.84 probability of correct identification. This parameter is an indicator of the participant’s sensitivity to relative change in motion and is therefore arbitrarily taken as the differential threshold. Normally, the probability of correct discrimination corresponding to a stimulus change of one differential threshold is arbitrarily chosen by the experimenter between 70 and 85 % (MacNeilage et al. 2010; Mallery et al. 2010; Naseri and Grant 2012).

In the baseline condition, the chance level of correctly detecting the amplitude discrepancy is at 0.5. Therefore, the inflection point of the cumulative Gaussian is located at 0.5 × (1 − 0.5) + 0.5 = 0.75 (see Fig. 4a). The corresponding motion amplitude was considered as the absolute threshold for the perception of linear vertical motion (Soyka et al. 2011, 2012).

A Bayesian adaptive procedure, based on the method proposed by Kontsevich and Tyler (1999), was used to estimate the psychometric function (Tanner 2008). The basic idea behind the method is to fit a psychometric function to the whole data set after each trial. Simulating the answer of the next trial for each possible comparison stimulus determines which stimulus would minimize the spread of the parameters’ posterior distribution according to an entropy-based cost function. Smaller entropies indicate higher confidence that the fitted psychometric function resembles the model underlying the participant’s behavior. The selected stimulus is considered the most informative and is used in the next trial. Making use of this method allows for a fast and accurate estimation of the psychometric function. In all but the baseline conditions, the efficiency of this method is increased by fixing the mean of the psychometric function to the pedestal stimulus value because there is one less free parameter to consider. Note how this allows for the method to select stimuli in regions that are more informative for estimating the standard deviation.

For each condition, participants were tested for at least 100 trials and until the estimate stabilized. Our criterion for stabilization was that the fluctuation in the threshold estimate provided by the Bayesian adaptive method over the last 20 trials (i.e., highest minus lowest value) became smaller than 5 % of the highest value. If this criterion was not achieved after a maximum of 200 trials, the algorithm stopped and the last estimate was selected as the threshold. No session had to be terminated due to participant fatigue or sickness.

### Data analysis

**Fig. 5:**
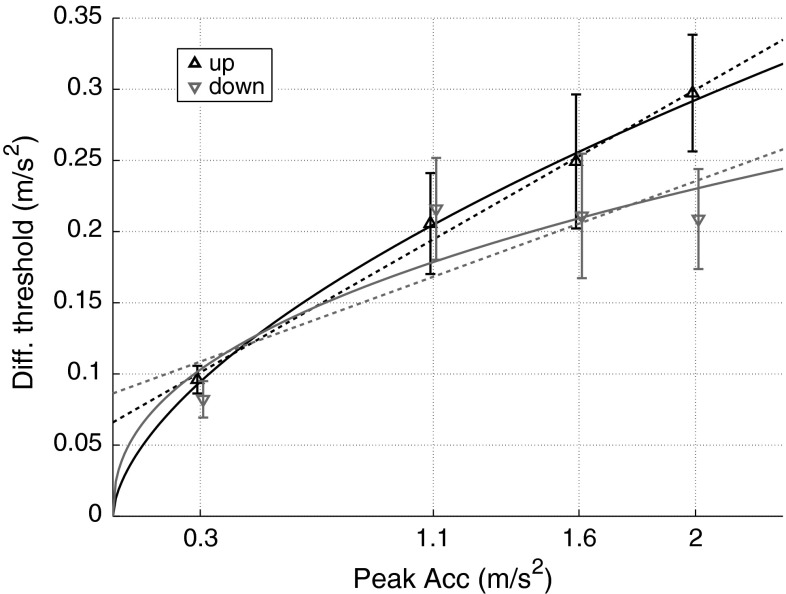
Differential thresholds for upward movements (*black triangles*) are significantly higher than for downward movements (*gray triangles*). *Error bars* are SEM. Their relationship with the motion intensity is more consistent with a power function (*black* and *gray continuous lines*) as opposed to a linear fit (*black* and *gray dashed lines*)

All analyses on the measured absolute and differential threshold estimates were performed in logarithmic units, but for convenience the averages are reported in m/s^2^ (see Fig. 5). The formulas used for the conversions are as follows:

3 $$abs\_th = e^{\alpha }$$

4 $$diff\_th = e^{{\left( {\alpha + \frac{\beta }{2}} \right)}} - e^{{\left( {\alpha - \frac{\beta }{2}} \right)}}$$

where *abs_th* and *diff_th* indicate the absolute and differential thresholds in linear space, respectively, and *α* and *β* are the mean and the standard deviation of the cumulative Gaussian, respectively, in logarithmical units.

To compare human sensitivity for upward versus downward motion at different pedestal intensities, data analysis was performed on the measured thresholds in logarithmic units. A repeated-measures ANOVA was used to test for significant differences between the 2 levels of the factor “motion direction” (upward and downward motion) and between the 4 levels of the factor “motion amplitudes”, corresponding to the different pedestal motion amplitudes between 0.3 and 2 m/s^2^. A paired samples *t* test was used to compare absolute thresholds for upward and downward motions. Linear and logarithmic threshold estimates and corresponding goodness of fit were compared with 2 repeated-measures ANOVA with 1 factor (“fit type”). Effects are considered to be significant if their *p* value is <0.05.

To establish the perceptual law governing human sensitivity to vertical self-motion, upward and downward data were fitted with three different models. The first model expresses Weber’s Law in its general form Δ*Φ* = *kΦ*, where *Φ* represents the stimulus intensity. A second model, suggested as an improvement to Weber’s Law, has the form Δ*Φ* = *k*(*Φ* + *a*), where *a* represents the amount of noise that exists when the stimulus is zero (Gescheider 1997). The third model is a power law (Δ*Φ* = *k* × *Φ* ^*p*^). The ability of these models to describe dependencies of upward and downward differential thresholds on motion intensity was quantified by the small-sample corrected version of the Akaike Information Criteria (AIC_c_) (Burnham and Anderson 2004), according to the formula:

5 $${\text{AIC}}_{\text{c}} = N\ln \left( {\frac{{\mathop \sum \nolimits_{i}^{N} \left( {y_{i} - f_{i} } \right)^{2} }}{N}} \right) + 2k + \frac{2k(k + 1)}{N - k - 1}$$

where *N* is number of data points, *k* is the number of model’s parameters, *y*_*i*_ represent the *i*th data point, and *f*_*i*_ is the model prediction for the stimulus associated with *y*_*i*_. AIC_c_ provides a relative measure of the models’ quality, assigning smaller values to models with a better trade-off between accuracy (prediction errors on the dataset) and complexity (high number of parameters).

## Results

A typical run required 133 trials on average and lasted approximately 40 min. In only one case did the algorithm fail to converge before 200 trials, otherwise the maximum number of trials required for one condition was 187.

Individual psychometric functions were fit in logarithmic space for each tested condition. The choice of fitting in the logarithmic space (Soyka et al. 2011, 2012) or in the linear space (Roditi and Crane 2012; MacNeilage et al. 2010) is arbitrary and, to the best of our knowledge, has never been systematically addressed. Data fitted in both domains are reported in Table 1. No significant differences were found between the goodness of fits [*F*(1, 91) = 1.27, *p* = 0.26] nor between threshold estimates [*F*(1, 91) = 0, *p* = 0.95]. We chose to analyze the data obtained with the logarithmic fit for two reasons. First, the adaptive procedure relied on an online logarithmic fit of the psychometric function to select the most informative stimuli. Additionally, the concept of fitting in the logarithmic space is consistent with decreased sensitivity for increasing motion intensities reported here and elsewhere (Mallery et al. 2010; Naseri and Grant 2012; Zaichik et al. 1999).

**Table 1:** Absolute and differential thresholds and corresponding goodness of fit (mean ± standard deviation) as obtained by fitting psychometric functions in the linear (“Lin” column) and logarithmic (“Log” column) domain. The latter are presented transformed according to Eqs. 3 and 4 to allow for comparison

Pedestal (m/s^2^)	Threshold (m/s^2^)		Entropy (bit)	
	Lin	Log	Lin	Log
Up				
0	0.066 ± 0.023	0.066 ± 0.023	2.963 ± 0.041	2.980 ± 0.195
0.3	0.118 ± 0.072	0.096 ± 0.031	2.413 ± 0.440	2.863 ± 0.246
1.1	0.209 ± 0.107	0.206 ± 0.112	2.702 ± 0.253	2.628 ± 0.275
1.6	0.231 ± 0.106	0.249 ± 0.149	2.764 ± 0.217	2.520 ± 0.325
2	0.286 ± 0.103	0.297 ± 0.130	2.875 ± 0.207	2.503 ± 0.298
Down				
0	0.069 ± 0.035	0.068 ± 0.033	3.039 ± 0.098	3.036 ± 0.247
0.3	0.095 ± 0.063	0.082 ± 0.040	2.224 ± 0.466	2.764 ± 0.178
1.1	0.216 ± 0.101	0.216 ± 0.113	2.720 ± 0.288	2.644 ± 0.311
1.6	0.198 ± 0.096	0.211 ± 0.138	2.658 ± 0.163	2.423 ± 0.326
2	0.210 ± 0.111	0.209 ± 0.111	2.704 ± 0.270	2.242 ± 0.311

Figure 5 shows that differential thresholds for vertical motion depend on pedestal amplitude [*F*(3, 27) = 54.2, *p* < 0.001] as well as on movement direction [*F*(1, 9) = 6.43, *p* = 0.009]. Asymmetries between upward and downward sensitivity seem to increase with stimulus intensity, although the interaction between motion direction and amplitude is not significant [*F*(3, 27) = 1.55, *p* = 0.22].

The absolute detection thresholds obtained with a pedestal acceleration of 0 m/s^2^ were 0.065 m/s^2^ for upward movements and 0.067 m/s^2^ for downward movements and not significantly different [*t*(5) = 0.033, *p* = 0.975]. This last finding is consistent with previous works on detection thresholds (Melvill Jones and Young 1978) and direction discrimination thresholds (Roditi and Crane 2012).

The Weber’s Laws that best fitted the collected data had coefficients *k*_up_ = 0.16 and *k*_down_ = 0.13 (AIC_c-up_ = −172.41, AIC_c-down_ = −172.25). For the improved Weber’s Law models (Δ*Φ* = *k*(*Φ* + *a*)), we obtained *k*_up_ = 0.12, *a*_up_ = 0.56,* k*_down_ = 0.07, and *a*_down_ = 1.16 (AIC_c-up_ = −173.09, AIC_c_down_ = −175.10). Finally, fitting the data with two power functions (Δ*Φ* = *k × Φ* ^*p*^) led to the coefficients *k*_up_ = 0.19, *p*_up_ = 0.60, *k*_down_ = 0.17, and *p*_down_ = 0.42 (AIC_c-up_ = −173.17, AIC_c-down_ = −176.39; Fig. 5, solid lines). For both upward and downward directions, the power law model reported the lowest AIC_c_ scores and is therefore the best candidate to represent the collected differential thresholds for vertical translations.

## Discussion

We investigated human sensitivity to vertical translations by independently measuring upward and downward differential thresholds for self-motion perception. These thresholds were found to be lower for downward compared to upward translations and to overall increase with stimulus intensity up to 0.3 m/s^2^ at a pedestal upward acceleration of 2 m/s^2^. According to AIC_c_, this trend is best described by a power law with exponents of 0.60 and 0.42 for upward and downward motion, respectively. We, however, point out that differences in AIC_c_ scores between the three compared models are often smaller than 2, suggesting only modest preference for the power laws (Burnham and Anderson 2004). The use of a power law to describe differential thresholds is attributed to Guilford (1932) and should not be confused with Stevens’ power law, which rather relates magnitude estimation responses to physical stimulus intensities. Notably, despite clear changes in motion sensitivity over the wide tested range, fitting psychometric functions in the logarithmic rather than in the linear space does not improve the quality of the fit. This suggests that, for small changes in motion amplitude, differential thresholds can be considered constant.

### Upward and downward sensitivity

We find a significant difference between differential thresholds for upward and downward motion, a difference that increases with stimulus intensity. This difference is not present at the level of the vestibular afferents, where cells preferentially excited by upward translations show comparable sensitivities with those preferentially excited by downward translations (Jamali et al. 2009). It is legitimate to consider whether asymmetries in the perception of vertical motion are an immediate consequence of the nonlinear perceptual law. Since the perception of vertical movements modulates around gravity (*G*), the net acceleration felt by human inertial sensors during downward acceleration is smaller (*G* − acc) than the one felt during upward acceleration of equal intensity (*G* + acc). Consequently, monotonically increasing perceptual laws (such as Weber’s law or the power law) predict thresholds for downward motion to always be smaller than any threshold for upward motion. However, arranging measured differential thresholds according to the net accelerations (from *G* − 2 to *G* + 2 m/s^2^) does not allow us to fit any constant or monotonic perceptual law. Decreasing sensitivities for net accelerations diverging from gravity lead to the conclusion that the brain compensates for gravity.

Having ruled out this explanation, there are at least three other factors that could explain the asymmetry in the perception of vertical translation. First, asymmetries might derive from central processing stages and cognitive factors. For instance, a higher weight could be assigned by the brain to the perception of downward motion given its importance for detecting falls and maintaining balance. A second explanation might be related to the noise introduced by the simulator on the commanded accelerations. In fact, downward movements of the CyberMotion Simulator present a higher signal-to-noise ratio than upward movements (Nesti et al. 2013). This means that a downward acceleration contains less noise as opposed to a vertical acceleration of equal commanded intensity, but it is unclear whether this difference is reflected in behavioral measures. A motion analysis of the simulator employed in Jamali et al. (2009), where comparable saccular afferent sensitivity is shown for cells responding to upward and downward motion, might help clarify this point. Finally, a third explanation for the effect of motion direction on motion sensitivity may reside in a significant contribution of somatosensory cues (Seidman 2008; Seidman et al. 2009). Indeed, during surge and heave, participants usually experience asymmetric tactile stimuli according to the direction of motion, whereas sway movements act symmetrically on the human body. Asymmetries in absolute detection thresholds for heave (in head coordinates) and surge are reported from Benson et al. (1986), with footward movements and backward movements being correctly detected more frequently than headward movements and forward movements, respectively. We speculate that, in our work, the use of foam padding might have reduced tactile cues during absolute thresholds measures up to the point where no significant evidence of their contribution on motion detection sensitivity could be observed. Nevertheless, the slower decrease in sensitivity that we observed for downward versus upward movement at higher motion intensities might reflect the influence of somatosensory cues.

These three alternatives can only be partially resolved by testing head-vertical sensitivity in the earth horizontal plane. Asymmetries are expected to disappear if they are derived from a balance mechanism or from different simulator-dependent noise, since balance is not threatened by horizontal motion in world coordinates and the simulator’s noise for horizontal motion is expected to be independent of motion direction. On the other hand, if asymmetries arise entirely or partially from asymmetric somatosensory cues, they may persist for head-vertical motion in the earth horizontal plane.

The importance of vestibular relative to proprioceptive and somatosensory information for body orientation and self-motion perception has been addressed by previous studies. In animals, extremely similar balance disorder and motor incoordination were observed by Carpenter et al. (1959) in labyrinthectomized cats and by Cohen (1961) in monkeys and baboons deprived of neck proprioceptors. This indicates equally essential roles of head-to-space information (arising from the vestibular system) and of head-to-trunk information (arising from neck proprioceptors). These questions have been addressed also in human vestibular loss patients (Cutfield et al. 2011; Gianna et al. 1996; Mallery et al. 2010; Valko et al. 2012; Walsh 1961; Agrawal et al. 2013), who in theory can only rely on somatosensory and proprioceptive cues. Overall, differences between healthy participants and patients show high variability, suggesting that the amount of information coming from non-vestibular sources varies with the experimental conditions, experimental setup, and motion profiles. For instance, Valko et al. (2012) showed thresholds from 1.3 to 56.8 times higher for vestibular loss patients than for healthy participants, depending on the frequency and the type of motion. Although it seems that the vestibular system plays a primary role in perceiving self-motion, from the observation that vestibular loss patients are still able to perceive motion in darkness we can safely conclude a contribution of the somatosensory and proprioceptive cues. This might explain asymmetric vertical sensitivity for upward and downward motion if these cues, asymmetric for heave motion, are noticeably different.

### Vertical self-motion sensitivity

Two power laws well describe the increase in upward and downward differential thresholds upon stimulus intensity (Fig. 5). In contrast, such a nonlinear dependence on motion intensity is not observed over the tested range by the saccular afferent fibers of the vestibular system in squirrel monkeys and rhesus monkeys (Fernández and Goldberg 1976b; Jamali et al. 2009) nor in human eye movements, which maintain a constant level of accuracy and precision over a wide range of rotation intensity (Pulaski et al. 1981; Weber et al. 2008). Perceptual nonlinearities must therefore arise at a further stage along the neuronal path that process self-motion information and are perhaps due to central processing of the vestibular signals, multisensory integration processes, and/or cognitive factors. The discrimination capability for high stimulus intensities is remarkable compared to other human sensory systems which are often best characterized by exponents close to 1 (Teghtsoonian 1971), i.e., the linear relationship between stimulus intensity and differential threshold described by Weber’s Law. As suggested by Mallery et al. (2010), the deviation from Weber’s law due to heightened sensitivity at larger stimulus intensities may be related to the role of the vestibular system for maintaining posture even at these high stimulus intensities.

Note that, for downward movements, one could almost argue that differential thresholds are independent of stimulus intensity, as only for the 0.3 m/s^2^ pedestal was the threshold significantly different than those for the other pedestals (Fig. 5). However, we still favor the power law over a conclusion of stimulus-independent downward sensitivity for several reasons. First, the data consistently show a clear difference at 0.3 m/s^2^ and confirm the differential threshold reported in MacNeilage et al. (2010). Second, it is more parsimonious to describe upward and downward sensitivity with the same function, which also leads to better AIC_c_ scores (AIC_c-down_ = −170.07 when fitting downward differential thresholds with their mean). Finally, dependencies are to be expected based on previous studies on self-motion sensitivity (Zaichik et al. 1999; Mallery et al. 2010; Naseri and Grant 2012).

A technical consideration is, however, necessary: for the motion simulator used here, as well as for other common motion simulators (Working Group AGARD, NATO 1979), the quality of the reproduced signals, expressed in terms of signal-to-noise ratio (SNR), increases with motion intensity (Nesti et al. 2013). Thus, it is possible that some amount of change characterized here by the exponents of the perceptual laws actually reflects variation in stimulus quality. Note that this is likely to be a very general problem, not specific to the current study. Similar SNR is expected for other simulators as well. However, none of the physiological studies mentioned above (Fernández and Goldberg 1976b; Jamali et al. 2009; Weber et al. 2008) reported changes in sensitivity, suggesting that changes in the simulator SNR over the tested motion range are not picked up by the vestibular system.

Heave differential thresholds have been investigated in the past by MacNeilage et al. (2010) and Zaichik et al. (1999). In the first study, a 6-degrees-of-freedom motion platform was used to generate 1-Hz sinusoid-like acceleration profiles. The differential threshold for a pedestal of 0.3 m/s^2^, measured with a 2IFC experimental design similar to the one we employed is reported to be 0.117 ± 0.078 m/s^2^. They also measured a direction discrimination threshold of 0.097 ± 0.034 m/s^2^ for vertical motion in a single interval discrimination task, where participants were asked to correctly identify the direction of motion (upward or downward). This value has to be multiplied by $\sqrt 2$ before comparing it with the results of a 2-interval discrimination task. Further accounting for the different threshold definition (84 % rather than 75 % correct answer probability), we obtain a comparable absolute threshold of 0.093 m/s^2^. Given the procedural similarities between this and the present study, it is possible to quantitatively compare our measurements with MacNeilage et al. (2010). Results agree for the differential thresholds at 0.3 m/s^2^, but our detection threshold of 0.066 m/s^2^ is lower than their direction discrimination threshold by approximately 30 % after correcting for the different definitions of absolute threshold (84 % rather than 75 % correct answer probability). This discrepancy can likely be attributed to the different experimental task. Indeed, thresholds are known to be higher for motion direction discrimination rather than motion detection, especially in the vertical direction (Melvill Jones and Young 1978). In Zaichik et al. (1999), a very different methodology was employed and a comparison of the measured thresholds would be meaningless. However, the Weber’s perceptual law they found for vertical movements in the range of 0–0.6 m/s^2^ is qualitatively consistent with our findings.

Absolute threshold can be defined as the differential threshold relative to a pedestal of zero. Unfortunately, the absolute thresholds measured in this study cannot be directly compared with differential threshold measured for non-zero pedestal values because of differences in the experimental design. However, if comparable methods had been used, we would expect the differential threshold for 0 m/s^2^ pedestal to be equal to or greater than the absolute threshold reported here. This would represent a significant deviation from the power law fits illustrated in Fig. 5. In fact, it would not be unreasonable to expect a decrease (or dip) in differential thresholds as pedestal increases from zero to small non-zero values, before increasing again with increasing pedestal. Such “dipper functions” are commonly observed in other psychophysical domains (Solomon 2009). The shape of the differential threshold curve from pedestal 0–0.3 m/s^2^ represents an interesting topic for future research.

Results from many psychophysical studies (e.g., Benson et al. 1986; MacNeilage et al. 2010; Roditi and Crane 2012; Zaichik et al. 1999; Valko et al. 2012; summarized in Table 2) agree that absolute thresholds are about 2 times higher for vertical motion than for horizontal motion. Absolute thresholds for horizontal linear motion have been previously investigated by Soyka et al. (2011) using the same simulator and a very similar setup. Although they did a direction discrimination rather than a detection task and at different frequencies (0.17, 0.42, 0.67 Hz), a comparison is still possible using the model they propose to account for frequency dependencies. Here, a direction discrimination threshold of about 0.02 m/s^2^ is predicted for horizontal sinusoidal acceleration profiles at 1 Hz, a value lower than the detection threshold we measured for vertical motion (0.066 m/s^2^), providing further evidence that humans are less sensitive to vertical motion.

**Table 2:** Absolute thresholds for 3D translations. Differences between measures are due to the task and the different simulators employed (Nesti et al. 2013), but within studies thresholds are consistently higher for vertical movements. Model prediction based on Soyka et al. (2011) and our results are grouped since the same simulator was employed

	Task	Stimuli	Threshold (m/s^2^)		
			Surge	Sway	Heave
Benson et al. (1986)	Discrimination	0.33-Hz sinusoidal acc	0.06	0.06	0.15^a^
Zaichik et al. (1999)	Detection	0.95-Hz sinusoidal acc	0.03	0.05	0.08
MacNeilage et al. (2010)	Discrimination	1-Hz sinusoidal acc	–	0.06	0.10
Roditi and Crane (2012)	Discrimination	1-Hz sinusoidal acc	0.03	0.03	0.08
Valko et al. (2012)	Discrimination	1-Hz sinusoidal acc	–	0.02	0.05
Soyka et al. (2011) (model)	Discrimination	1-Hz sinusoidal acc	0.02^b^	0.02^b^	–
Present data	Detection	1-Hz sinusoidal acc	–	–	0.07

^a^Participants were lying on their back, which MacNeilage et al. (2010) have shown raises perceptual thresholds

^b^Data were not significantly different for surge and sway and were therefore pooled before fitting the model

By comparing our results with Naseri and Grant (2012), we show that differences in sensitivity to horizontal and vertical motions exist not only at absolute threshold level but also for supra-threshold movements. Indeed, our data show differential thresholds for vertical movements that are always higher than those measured by Naseri and Grant (2012) for horizontal movements over the same motion range (up to 2 m/s^2^). The lower frequency used for their stimuli (0.25–0.6 Hz) supports this conclusion even more, since self-motion perceptual thresholds are known to decrease at higher frequencies (Benson et al. 1986; Soyka et al. 2011). It has been suggested that lower thresholds for horizontal than for vertical linear motion might derive from differences in the otolith organs response to linear acceleration acting on different axis of the head (Benson et al. 1986). Neurophysiologic measures of the primary afferent neurons in the squirrel monkey show sensitivity (spikes/s/g) about 30 % higher for horizontal than for vertical movements (Fernández and Goldberg 1976a). More recent neuronal findings (Jamali et al. 2009; Yu et al. 2012) report however similar gains for otolith afferents of the rhesus monkey when responding to horizontal and vertical translation. It is therefore not clear whether higher perceptual thresholds for vertical as compared to horizontal translations partially reflect a property of the vestibular afferents, but the perceptual discrepancies are anyway higher than what the neurophysiologic data from Fernández and Goldberg (1976a) would predict. This incongruence between objective and subjective data could again reside in higher level processing of the vestibular signals, in multisensory integration or in cognitive factors. As was suggested above, a possible interpretation for vertical asymmetries is that the central nervous system might favor horizontal sensitivity more than vertical sensitivity since it is more informative for balance control.

## Footnotes

1 All the probabilities reported in this paragraph might be slightly affected by the lapse parameters.
